# Burden and Experiences of Head Lice Infestation Among Children in Western Australia

**DOI:** 10.1155/japr/8631800

**Published:** 2026-03-05

**Authors:** Tina Barrow, Stephanie Enkel, Hannah Thomas, Ingrid Amgarth-Duff, Tracy McRae, Lorraine Anderson, Julie Marsh, Rachel Burgess, Rebekah Newton, Asha Bowen

**Affiliations:** ^1^ Wesfarmers Centre of Vaccines and Infectious Diseases, The Kids Research Institute, Perth, Western Australia, Australia; ^2^ School of Medicine, The University of Notre Dame Australia, Fremantle, Western Australia, Australia, nd.edu.au; ^3^ Western Australian Country Health Service, Government of Western Australia, Perth, Western Australia, Australia, wa.gov.au; ^4^ Medical School, The University of Western Australia, Perth, Western Australia, Australia, uwa.edu.au; ^5^ School of Biomedical Sciences, Microbiology and Immunology, The University of Western Australia, Perth, Western Australia, Australia, uwa.edu.au; ^6^ Kimberley Aboriginal Medical Services, Broome, Western Australia, Australia; ^7^ Royal Perth Hospital, Perth, Western Australia, Australia, rph.wa.gov.au; ^8^ Department of Infectious Diseases, Perth Children′s Hospital, Nedlands, Western Australia, Australia

**Keywords:** head lice, impetigo, *Pediculus humanus capitis*, scabies, tinea

## Abstract

**Background:**

Head lice is an ectoparasitic skin infection commonly seen in primary school–aged children. In remote Australia, where rates of other skin infections and downstream sequelae are endemic, the rate of head lice infestation is unknown.

**Methods:**

This multimethod observational study is aimed at describing the burden of head lice for remote‐residing children from nine communities in the Kimberley, Western Australia. Qualitative and quantitative data collected by the See, Treat, Prevent Skin Sores and Scabies (‘SToP’) Trial at 10 timepoints between May 2019 and December 2022 were analysed to understand head lice rates and community perspectives.

**Findings:**

Across the Kimberley over the course of the study, the mean head lice prevalence was 48.4% (SD 15.3). Of children with head lice detected at any timepoint (*n* = 554), repeated detection occurred at a rate of 66.8% (370/554). Head lice was mentioned in 42 yarning sessions with community members and service providers. Community voice reflected repeated head lice infestation to be detrimental to the physical and psychological well‐being of children. Much of the discussion focussed on prevention of secondary bacterial infections and improving the quality and maintenance of housing to provide for healthy practices to reduce the rates of head lice.

**Conclusion:**

In remote‐residing Australian Aboriginal children, there is a high burden of head lice. Community members reflected the impact of head lice on children′s well‐being and focused on addressing contributing environmental factors and preventing secondary bacterial infection.

**Trial Registration:**

Australian New Zealand Clinical Trials Registry Number: ACTRN12618000520235

Statement of Respect

Throughout this paper, Aboriginal and Torres Strait Islander peoples are respectfully referred to as Aboriginal, as this is the preferred terminology in Western Australia where this project was conducted. We recognise and acknowledge the diversity between Aboriginal and Torres Strait Islander cultures across Australia, and do not intend to diminish any identity. We respectfully acknowledge Aboriginal ownership of the land, waters and sky of Australia in continuity for more than 65,000 years.

## 1. Introduction

Head lice (*Pediculus humanus capitis)* are hematophagous ectoparasites that pose a significant and costly global public health issue [1], identified both as a neglected tropical disease (NTD) and listed on the World Health Organization (WHO) priority list for elimination [2].

Head lice can appear as eggs (nits) attached to the hair shaft or as live lice (Figure 1). The pruritis, arising from mite salivary antigens, can take weeks to develop [3]. Infestations commonly affect primary school–aged children, spreading easily via head‐to‐head contact in busy classrooms and playgrounds [4]. Infection with head lice can disrupt sleep, impact self‐esteem, reduce school attendance and lead to iron‐deficiency and anaemia [5, 6]. Current preventative strategies include regular inspection (particularly around the nape of the neck and behind the ears), repellents such as citronella essential oil and limiting head‐to‐head contact [3]. In Australia, clinical guidelines recommend the first‐line use of topical treatments such as pyrethrin, dimeticone or malathion; mechanical removal; treating close contacts and hot‐washing and sun‐drying linen [3]. For refractory head lice or when topical treatment is unavailable, oral ivermectin is recommended as a second‐line option [3, 7, 8]. The use of oral ivermectin as a first‐line agent has been proposed due to greater effectiveness, proven safety and ease of oral administration over a topical agent; however, it is currently not licensed for head lice in Australia [9].

Figure 1Examples of head lice infection in the hair of remote‐living Western Australian children. (a) Eggs (nits) seen on hair shafts close to the scalp. (b) Live lice seen on hair shafts further from the scalp.

**Figure:**
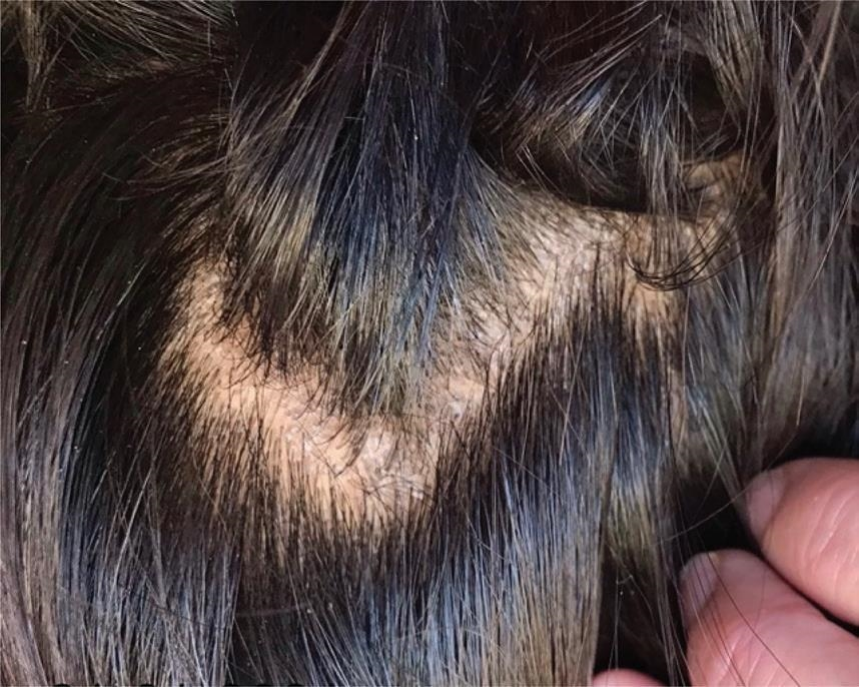
(a)

**Figure:**
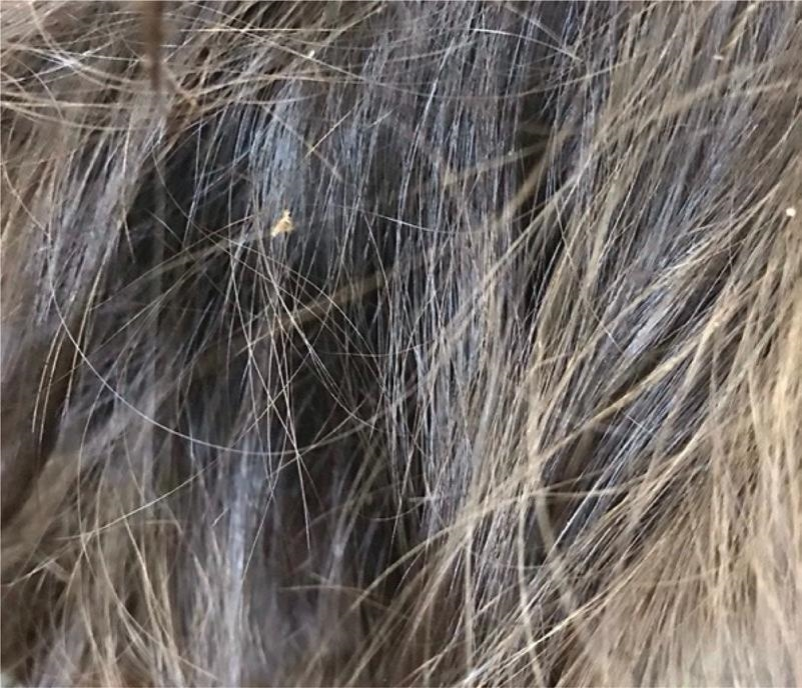
(b)

Compulsive scratching of the scalp in response to the itchiness of head lice infection can damage the skin barrier, creating an opportunity for harmful skin infections caused by *Group A Streptococcus* (Strep A) and *Staphylococcus aureus.* When inadequately treated, Strep A infections can develop into acute rheumatic fever (ARF), rheumatic heart disease (RHD) or acute poststreptococcal glomerulonephritis [10, 11]. Remote‐residing Australian Aboriginal children are disproportionately burdened by Strep A infection and experience endemic rates of RHD alongside impetigo and scabies [10, 11]. Head lice affect children in all socioeconomic circumstances, but the highest estimates of prevalence (approximately 40%) are seen in low‐resourced settings [12]. The burden of head lice and the impacts of this infection on remote‐residing Australian Aboriginal children have not yet been described.

In this multimethod observational study, we are aimed at describing the burden and impact of head lice for remote‐residing Australian Aboriginal children. During the conduct of a clinical trial focused on impetigo and scabies in the Kimberley region of Western Australia between 2019 and 2022, we collected additional data on head lice infestations during routine skin checks. The data reported offers an estimate of head lice prevalence in the remote Australian setting, alongside qualitative findings from interviews and yarning sessions exploring the perspectives and priorities of community members regarding head lice.

## 2. Methods

### 2.1. Positionality Statement

Collectively, we the authors identify as a cross‐cultural team, and each brings our own unique worldviews to this research. A commitment to practising reflexivity provided a space where we, as researchers, examined our own worldviews and assumptions of positionality within the research context. Adopting a critical reflexive lens, and from working in two‐way learning partnerships with Aboriginal communities, we acknowledge and centralise Aboriginal ways of knowing, doing and being throughout this research [13, 14].

### 2.2. Study Design

This multimethod study utilises quantitative and qualitative data that were previously collected during the See, Treat, Prevent Skin Sores and Scabies (SToP) Trial: a cross‐sectional, cluster randomised, open cohort, stepped‐wedge trial involving nine remote communities in the Kimberley, Western Australia. The SToP Trial is registered with the Australian New Zealand Clinical Trials Registry, Number ACTRN12618000520235. The primary outcomes and evaluation of the trial have been published elsewhere [11, 15]. In short, participating communities were pragmatically invited to participate based on recommendations from the Kimberley Aboriginal Health Planning Forum and in consultation with community elders. Communities were designated into four geographically distinct clusters (Figure S1), and the clusters were randomised into two steps that progressively participated in interventional activities aimed at decreasing the rates of impetigo and scabies infection [16].

Head lice data were collected throughout the SToP Trial. A focus on head lice was not determined a priori, and as such no activities to address head lice were protocolised and no data were collected to confirm whether treatment was administered following initial detection. Hence, an observational cohort study within a clinical trial is described. Data were collected at 10 timepoints between May 2019 and December 2022. Outside the May–December periods, the ‘wet season’ compromised safe access to remote communities due to regional flooding and subsequent damage to roads (Figure S2). Two scheduled timepoints in April–June and July–September 2020 did not proceed due to COVID‐19 pandemic–related restrictions, and the project was extended for a further two timepoints.

### 2.3. Study Population and Setting

The Kimberley is one of the most remote regions in WA—it has an estimated population of 35,000 people with a median age of 33 years, of whom 41.1% are Aboriginal Australians [17]. Study participants included children who participated in school‐based skin checks at least once (quantitative data) and community members aged 18 years or older who participated in one or more semistructured interviews/yarning sessions (qualitative data).

### 2.4. Data Collection

#### 2.4.1. Qualitative

Participants provided written informed consent prior to participation. Semistructured interviews and yarning sessions were conducted face‐to‐face by Aboriginal and non‐Aboriginal study researchers. All researchers had prior interviewing experience, and were provided with cultural training and support from the Kulunga Aboriginal Unit at The Kids Research Institute Australia. Participants were recruited through both purposive and snowball sampling as previously described [11]. The use of yarning in qualitative research was formalised by Aboriginal academic, Dawn Bessarab, where semistructured conversations with Australian Aboriginal participants prioritise respectful sharing of cultural knowledge [18]. Aboriginal participants have ownership and leadership in research yarning sessions, and the researcher actively listens and collaboratively learns [19]. The research yarn supports Aboriginal ways of knowledge sharing rather than Western social science methodologies [18]. In this study, emphasis was placed on building relationships and establishing rapport as a key aspect of yarning methodology, and yarning circle participant numbers were determined by the community members present. The qualitative methodology of the broader SToP Trial has been described in detail previously [15].

#### 2.4.2. Quantitative

Written informed consent was obtained from caregivers to conduct skin checks for children aged 0–18 years. Children provided verbal assent prior to each skin check. At each of the 10 study timepoints, trained researchers examined the exposed skin of all present, consented and assenting children and systematically documented the presence or absence of impetigo, scabies, tinea and head lice per child and body site [16]. These skin infections were diagnosed based on characteristic clinical findings, as described previously [16]. Head lice cases were defined as the presence of head lice or eggs detected on visual inspection of the scalp. Head lice coinfection was defined as the presence of another skin infection, such as tinea, impetigo or scabies, on the scalp of a child with head lice. All data were initially recorded on a standard deidentified paper case report form and subsequently entered into Medrio—a secure electronic database [20]. As head lice was not a prespecified outcome of the SToP Trial, no protocolised follow‐up was conducted to determine whether treatment was administered after a detection, nor to confirm resolution prior to subsequent visits. As such, repeated detections across visits reflect *repeat presentations* rather than confirmed reinfection, and persistent unresolved infestations cannot be distinguished from true reoccurrences.

### 2.5. Statistical Analysis

#### 2.5.1. Qualitative

A combination of deductive and inductive thematic analysis was independently conducted by two research staff with qualitative experience (T.B. and S.E.). To ensure rigour, preliminary themes were independently identified and refined collaboratively until the final themes and subthemes were established. Two additional researchers (H.T. and I.A.D.) checked the themes and contributed further interpretation when required. Transcripts were managed and analysed using NVIVO QSR International Pty Ltd Version 12 software package (2022) [21].

#### 2.5.2. Quantitative

Analyses were conducted using R Studio [22] and SPSS [23]. Rates of infection were calculated as proportions at each timepoint and reported as means with standard deviation for the four clusters at each of the 10 timepoints. D′Agostino and Pearson test analysed normality of data, where data were normally distributed, a one‐way ANOVA using Tukey′s multiple comparisons test was employed to determine significance. Data that were not normally distributed were analysed with the Kruskal–Wallis test.

### 2.6. Ethics

This observational study reports data collected during the SToP Trial, with support from the Kimberley Aboriginal Health Planning Forum Research Subcommittee (2017–2018) and ethical approval from the Child and Adolescent Health Service (RGS0000000584) and Western Australia Aboriginal Health Ethics Committee (Approval Number 819). Approval was also provided by the University of Western Australia (RA/4/20/4123), Catholic Education Western Australia (RP2017/57), Western Australia Department of Education (D18/0281633), University of Notre Dame (Reference Number 2021–128F) and Murdoch University (2022/196) for student projects.

### 2.7. Role of the Funding Source

The funders had no role in study design, data collection, analysis, interpretation or preparation of the manuscript.

## 3. Results

Over 4 years, 152 individuals—46 community members, 69 school staff members, 29 clinic staff members and eight other service providers—participated in interviews and yarning sessions about skin health. Of these, 124 participants provided consent for audio recording and transcription, whereas the remaining sessions were recorded through notetaking. The topic of head lice was raised and discussed in 42 of these interviews and yarning sessions, forming the basis of the qualitative analysis.

Over the 10 study timepoints, 777 consented children had their skin checked for infections at least once. The demographic information of all children participating in skin checks at the first timepoint is provided as an exemplar of the study population (Table 1). Despite the open cohort study design, there were no significant variations in these proportions across the course of the trial—demographic information for all study timepoints has been published previously [11].

**Table 1:** Community cluster demographics at first timepoint (May 2019).

	Cluster A (*N* = 169)	Cluster B (*N* = 53)	Cluster C (*N* = 77)	Cluster D (*N* = 83)
Age (years)				
Median (Q1, Q3)	8.4 (6.2, 10.7)	9.3 (6.0, 12.0)	8.3 (5.6, 10.8)	8.7 (5.3, 11.0)
Height (cm)				
Median (Q1, Q3)	131.0 (117.0, 144.0)	137.0 (116.0, 152.5)	128.0 (110.8, 141.0)	130.0 (110.0, 145.0)
Missing	4	2	11	14
Weight (kg)				
Median (Q1, Q3)	25.3 (19.7, 36.0)	30.0 (19.0, 37.2)	25.1 (20.6, 40.5)	25.8 (18.7, 37.4)
Missing	4	2	11	14
Aboriginal				
Yes	157 (93%)	48 (91%)	72 (94%)	79 (95%)
No	11 (7%)	5 (9%)	5 (6%)	4 (5%)
Unknown	1 (1%)	0 (0%)	0 (0%)	0 (0%)
Gender				
Female	84 (50%)	25 (47%)	42 (55%)	39 (47%)
Male	84 (50%)	28 (53%)	35 (45%)	44 (53%)
Other	1 (1%)	0 (0%)	0 (0%)	0 (0%)

Participants who raised head lice as a topic of discussion reported high levels of infestation among children in their communities. Although many felt the high rates were an issue, some also acknowledged that head lice were perceived as a normal part of life.

*‘*I see it a lot, I guess it is normal. We grew up always having lice and dealing with lice, and it is not good. We know it is not healthy for the head*.’*

M2, community member.

*‘*Almost every child I have seen in the past couple weeks has had head lice out here.*’*

C013, clinic staff member.

**Figure 2:**
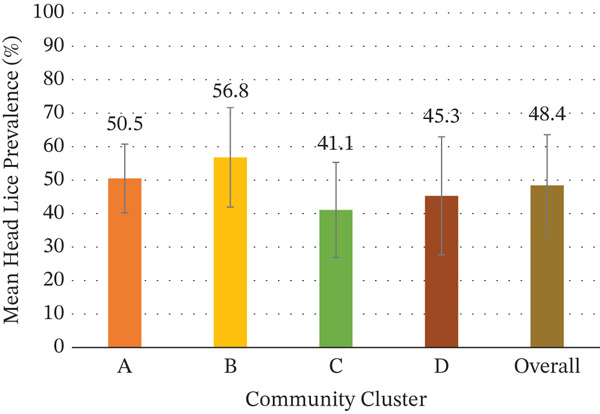
Head lice prevalence by community cluster (mean +/− SD). ‘Overall’ represents the mean prevalence across all clusters and visits.

These perceptions were supported by observational data, with 71% (554/777) of children who participated in skin checks found to have head lice at least once during the study period. The mean prevalence of head lice across all timepoints was 48.4% (SD 15.2) with a median prevalence of 47.0% (IQR 39.7–61.5) (Figure 2, Table S1). There was no significant variation in mean prevalence between the four geographically distinct study clusters.

Participants expressed frustration that children repeatedly experienced recurring head lice despite receiving treatment, with recurrent sentiments that the issue would be maintained regardless of efforts to continually treat.

‘With head lice, I think as a parent a lot of us parents try to get it under control, and then maybe a month later, we look at our child′s head and find a lot of head lice.’

M5, community member.

‘I reckon you cannot really like get rid of it, hey? Because it is always going to be there, and I know that is stupid and that is hard to deal with.’

M2, community member.

**Figure 3:**
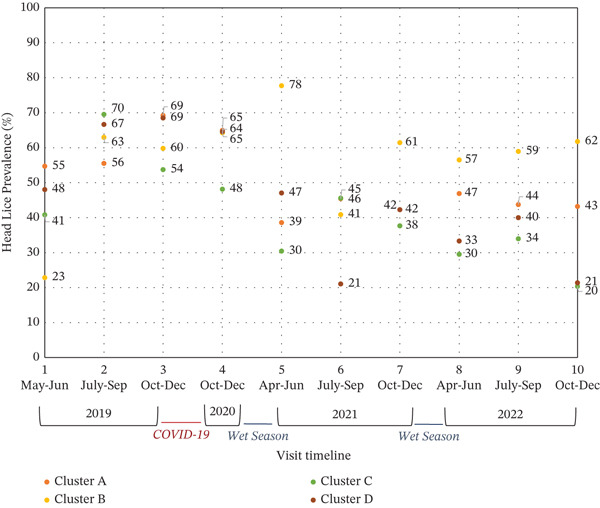
Head lice prevalence over the course of the study.

Consistent with participants′ perceptions, the prevalence of head lice did not change markedly over the course of the study. At the first timepoint in 2019, prevalence ranged from 23% to 55% across the four clusters (Figure 3). By the final timepoint in 2022, prevalence ranged from 21% to 62%.

Of the 554 children identified with head lice at least once during the study, 66.8% (370/554) had head lice again at a later visit. As treatment uptake and clearance were not assessed, these repeated detections represent repeat occurrences rather than confirmed reinfections.

Some clinic staff raised a link between head lice and other skin infections, describing damage to the skin barrier from scratching, leading to coinfection with bacteria and/or fungus. Further, more severe cases of impetigo were believed to be associated with head lice.

‘Impetigo, I feel like in particular can often be caused with lice… A lot of the worst impetigo I see is caused by head lice.’

C031, clinic staff member.

However, when examining all head lice presentations across all clusters at all timepoints (*n* = 1488), only 2.3% (SD 2.5) were associated with impetigo of the scalp. Co‐occurrence of head lice with other skin infections was more common, with simultaneous infection of scabies, tinea or impetigo (at any site on the body) at rates of 66.7% (SD 18.9), 55·1% (SD 13.3) and 54.2% (SD 10.9), respectively.

Beyond physical impacts, participants described the socioemotional effects of head lice. They noted that shame and stigma relating to head lice contributed to emotional distress among children. In school settings, head lice were thought to be linked to disrupted school attendance, reduced concentration with learning and an additional load for both the children and the teachers.

‘[…] they might feel shame, and then we might not see the children for a few days.’

S052, school staff member.

‘It is detrimental to their learning…I know personally I could not focus through that, they do an amazing job with that.’

S030, school staff member.

Some participants identified factors such as crowded living conditions and frequent movement between houses as contributors to head lice transmission. Improving environmental health and educating families were emphasised as important, yet unmet, needs to address the burden of head lice.

‘In reality, they will just catch it from another child because they move so much between houses.’

S046, school staff member.

‘[…] I reckon to educate family and homes about healthy environment, always having a clean yard, having a clean bed, having clean towels you know? Not sharing towels, which is a big thing that in our mob we share you know?’

M2, community member.

Community members spoke about using multiple modalities to treat head lice including medicated hair wash, nonmedicated soaps and shaving hair. However, these methods were largely perceived as ineffective.

‘A lot of people here you know they think sometimes the only way to do it is to shave the kid′s hair off.’

M4, community member.

‘Something I can see that families are really struggling to keep on top of their little child′s nits in their hair…they are struggling trying all sorts of treatment.’

Group S007, school staff member.

## 4. Discussion

This is the first study to report community perspectives on the burden of head lice for remote‐residing Australian Aboriginal children alongside quantitative prevalence data. Participants described a high burden of head lice and highlighted the impact of this on the well‐being of children and the wider community. Observational data corroborated this high burden, which did not meaningfully change from 2019 to 2022.

A meta‐analysis estimates the global burden of head lice to be 19% in school‐aged children [4], and head lice prevalence in urban‐living Western Australian Aboriginal children has previously been estimated to be 23% [24]. Here, we report a higher rate of head lice for remote‐residing Aboriginal children within the Kimberley region, with almost half of all skin checks documenting head lice and more than two thirds of the participating children presenting with head lice at least once across the study period. Community voices emphasised that this was an issue necessitating action, although some also felt that head lice were a normal part of childhood.

Previous work in WA has demonstrated that parents/caregivers, health practitioners and service providers experiencing prolonged periods of high rates of skin infections may become desensitised to and not consider skin infections to be a health concern or priority [25–27]. This phenomenon of ‘normalisation’ results in head lice and other skin infections going unnoticed, particularly where there are competing priorities and a lack of resources [28]. The SToP Trial delivered skin surveillance, referrals for treatment and community capacity building around skin health with a focus on environmental health [16]. Although the trial only specifically targeted impetigo and scabies, we must acknowledge that these skin health activities may have had the potential to combat the normalisation of skin infections more broadly, thus possibly influencing the head lice burden. We observed a minimal decline in head lice rates across the course of the study; however, the head lice burden at the end of the study remained high.

Children who had head lice experienced a high burden of other skin infections, with the vast majority having head lice more than once, many at consecutive visits, alongside higher rates of scabies, tinea and/or impetigo infections than children without head lice. Participants reflected that head lice predispose children to other skin infections, particularly impetigo. In remote communities of northern Australia it has been reported that scabies, head lice and impetigo frequently coexist [10]. As well as causing breaks in the skin, head lice feeding near impetigo lesions may act as a passive vector for *Staphylococci* and *Streptococci* [10, 12]. Shared social and environmental risk factors contributing to multiple skin infections may explain why the same children are impacted with frequent reinfection.

To the affected child, head lice infestations can have psychological and physical impacts. Community members and service providers described head lice infections causing stigma and shame, and impacting school engagement. This is consistent with impacts of head lice previously reported in other settings, with the associated shame and stigma potentially leading to delayed presentations and under‐treatment [5]. Community members reported treating head lice by shaving hair and using medicated hair wash, and they focused on hand hygiene to prevent bacterial infections. Topical solutions (permethrin, dimeticone or malathione) and lice combs are the current first‐line recommendations [3]. Challenges in topical solutions include children being unable to sit still for prolonged periods, availability of caregivers to spend hours on consecutive days combing hair, affordability, access to clean water and a well‐maintained bathroom and lice resistance [10, 11]. Shorter hair has been associated with lower rates of lice [28], although hair shaving may further impact self‐esteem for children. There may be cultural implications of shaving hair in remote Aboriginal communities, and further consultation to explore the impacts or benefits of this strategy to treat lice should be mindful of this. Overall, new treatment options may be of great benefit to the community.

Oral ivermectin can overcome many of the impracticalities and unaffordability of other topical solutions and work is underway to bring this into standard practice as a treatment for head lice [9]. A mass drug administration (MDA) of ivermectin for the treatment of scabies in a remote Aboriginal community in Australia successfully lowered the prevalence of scabies from 4% to 1%, although this reduction was short‐lived [29]. This study demonstrated that the benefits of an MDA are unlikely to be sustained without addressing the contributing social and environmental factors [29], and the need for environmental health support was resoundingly reinforced throughout our discussions about head lice. Any initiative in a remote Aboriginal community setting must be community‐led, codesigned and strength‐based with respect for reciprocity [15], and the importance of this cannot be understated. Community members in this study made it clear that no treatment efforts are likely to deliver sustained success without first addressing the environmental and housing factors that contribute to high burdens of infectious diseases in remote areas of Australia. Indeed, studies in Iran and India have demonstrated that factors such as lower levels of education, socioeconomic status and hygiene practices contribute to higher rates of head lice infections [28, 29]. These findings also carry important implications for health practice and policy. The persistently high burden of head lice, combined with community‐identified environmental contributors, highlights the need for sustained investment in environmental health, improved access to practical and effective treatment options and integration of head lice into broader remote public health planning. Ensuring that responses are community‐led and culturally grounded will be essential for any policy or programme aiming to reduce this burden.

## 5. Strength and Limitations

The large sample size of this dataset spans across 4 years and nine communities, lending reliability to the head lice prevalence observed. This head lice rate may be generalisable to other remote communities in northern Australia where environments, climate and burden of other related diseases are similar.

Data were collected only during school terms, when children are most near to each other, and this may have contributed to a higher overall estimated prevalence. It should be noted that data were not collected during the wet season, although there is no current evidence to suggest that head lice infections display seasonality. Not all children were able to participate at every visit and this limits analysis of persistent infection. ‘Reinfection’ rate could not be described as it is unknown if cases were treated between visits. Furthermore, head lice were detected by direct visualisation of the scalp/hair, and wet‐combing may be a more sensitive method.

The multimethod observational outcomes reported here enable some contextualisation of the quantitative data in the sociocultural landscape of participating remote communities. These interviews were designed around impetigo and scabies, and thus qualitative data pertaining specifically to head lice was limited. The qualitative findings reported are specific to these communities and those who participated in interviews and opinions described cannot be assumed to represent the perspective of every member of the community or communities elsewhere.

## 6. Conclusion

Here we report very high rates of head lice in remote communities of Australia, and that the harm of head lice extends beyond the itch. Holistic community‐led strategies are required to address this persistent public health concern and reduce the burden of head lice for remote‐residing Australian Aboriginal children.

## Funding

This study was supported by the Department of Health, Government of Western Australia, (10.13039/100031198) (FHWAYR3‐201516‐KHS); Australian National Health and Medical Research Council (RGS0000000584) and Healthway (10.13039/501100000960) Project 33088.

## Disclosure

WA Country Health Service and Kimberley Aboriginal Medical Service had no impact on study design, data collection and analysis, decision to publish or preparation of the manuscript.

## Conflicts of Interest

The authors declare affiliations with the WA Country Health Service and Kimberley Aboriginal Medical Service.

## Supporting information


**Supporting Information** Additional supporting information can be found online in the Supporting Information section. Figure S1: Participating community clusters located in the Kimberley, Western Australia. Figure S2: Timeline of the 10 community visits from May 2019 to December 2022. Yellow blocks represent the wet season, and the dark grey block represents cancelled visits during the Covid‐19 pandemic. Table S1. Head lice prevalence by community cluster and visit number.

## Data Availability

The participants of this study did not give written consent for their data to be shared publicly, so due to the sensitive nature of the research, supporting data is not available.
