# Supplementary material for: Burden and Experiences of Head Lice Infestation Among Children in Western Australia
Source: J Parasitol Res. 2026 Mar 5;2026:8631800. doi: 10.1155/japr/8631800 (PMC12961354; doi:10.1155/japr/8631800)
Supplement: Supplementary file 1 — Supporting Information Additional supporting information can be found online in the Supporting Information section. Figure S1: Participating community clusters located in the Kimberley, Western Australia. Figure S2: Timeline of the 10 community visits from May 2019 to December 2022. Yellow blocks represent the wet season, and the dark grey block represents cancelled visits during the Covid‐19 pandemic. Table S1. Head lice prevalence by community cluster and visit number. [file JAPR-2026-8631800-s001.docx]

## Supplementary material


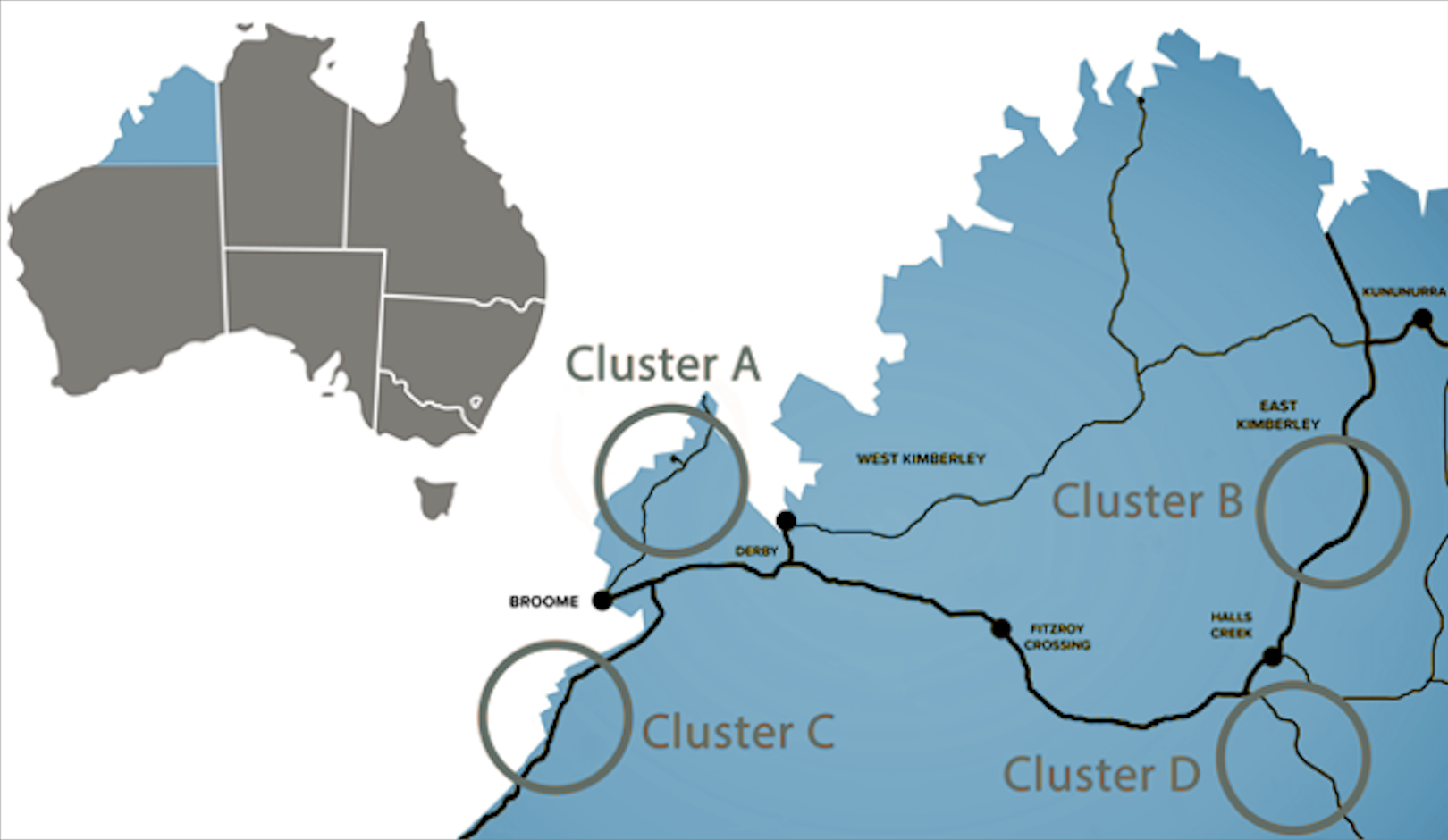


**Supplementary Figure 1. Participating community clusters located in the Kimberley, Western Australia.**


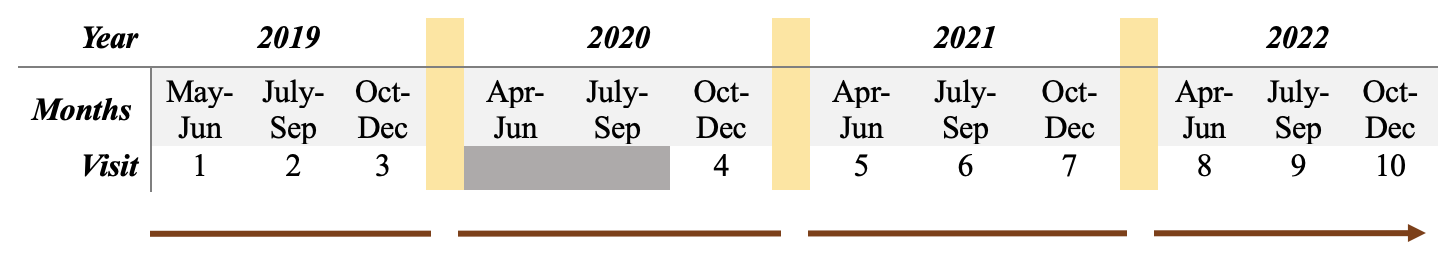


**Supplementary Figure 2. Timeline of the ten community visits from May 2019 to December 2022.** Yellow blocks represent the wet season, and the dark grey block represents cancelled visits during the Covid-19 pandemic.

**Supplementary Table 1. Head lice prevalence by community cluster and visit number.**

| **Visit** | **Cluster A**  n/N (%) | **Cluster B**  n/N (%) | **Cluster C**  n/N (%) | **Cluster D**  n/N (%) | **Overall**  Mean % (SD) |
| --- | --- | --- | --- | --- | --- |
| 1 | 29/53 (54·7) | 19/83 (22·9) | 69/169 (40·8) | 37/77 (48·1) | *41*·*8 (13*·*7)* |
| 2 | 20/36 (55·6) | 51/81 (63·0) | 112/161 (69·6) | 44/66 (66·7) | *64*·*0 (6*·*1)* |
| 3 | 27/39 (69·2) | 67/112 (59·8) | 79 /147 (53·7) | 37/54 (68·5) | *63*·*0 (7*·*3)* |
| 4 | 26/40 (65·0) | 45/70 (64·3) | 39/81 (48·1) | 42/65 (64·6) | *60*·*5 (8*·*3)* |
| 5 | 17/44 (38·6) | 77/99 (77·8) | 39/ 128 (30·5) | 24/51 (47·1) | *48*·*5 (20*·*9)* |
| 6 | 20/44 (45·5) | 27/66 (40·9) | 57/ 125 (45·6) | 12/57 (21·1) | *38*·*2 (11*·*7)* |
| 7 | 22/52 (42·3) | 51/83 (61·4) | 46 /122 (37·7) | 22/52 (42·3) | *45*·*8 (10*·*3)* |
| 8 | 23/49 (46·9) | 39/69 (56·5) | 34/115 (29·6) | 19/57 (33·3) | *41*·*8 (12*·*6)* |
| 9 | 21/48 (43·8) | 46/78 (59·0) | 34/100 (34·0) | 24/60 (40·0) | *44*·*3 (10*·*7)* |
| 10 | 16/37 (43·2) | 42/68 (61·8) | 21/103 (20·4) | 12/56 (21·4) | *36*·*5 (20*·*0)* |
| **Overall**  Mean % (SD) | *50*·*5 (10*·*3)* | *56*·*8 (14.8)* | *41*·*1 (14*·*2)* | *45*·*3 (17*·*6)* | *48*·*4 (15*·*2)* |
